# Simultaneous dual-energy X-ray stereo imaging

**DOI:** 10.1107/S1600577515006554

**Published:** 2015-06-26

**Authors:** Rajmund Mokso, Peter Oberta

**Affiliations:** a Paul Scherrer Institute, Swiss Light Source, CH 5232 Villigen, Switzerland; b Institute of Physics of the Academy of Sciences of the Czech Republic, v.v.i., Na Slovance 1999/2, Praha 8, Czech Republic; c Rigaku Innovative Technologies Europe s.r.o., Novodvorska 994, Praha 4, Czech Republic

**Keywords:** optics, crystals, imaging, dual-energy

## Abstract

Dual-energy or *K*-edge imaging is used to enhance contrast between two or more materials in an object and is routinely realised by acquiring two separate X-ray images each at different X-ray wavelength. On a broadband synchrotron source an imaging system to acquire the two images simultaneously was realised. The single-shot approach allows dual-energy and stereo imaging to be applied to dynamic systems. Using a Laue–Bragg crystal splitting scheme, the X-ray beam was split into two and the two beam branches could be easily tuned to either the same or to two different wavelengths. Due to the crystals’ mutual position, the two beam branches intercept each other under a non-zero angle and create a stereoscopic setup.

## Abstract 2

A Laue–Bragg geometry is introduced for splitting an X-ray beam and tuning each of the two branches to selected wavelength. Stereoscopic and dual-energy imaging was performed with this system.

## 1. Introduction   

When an X-ray beam traverses a medium both the real and the imaginary part of the complex refractive index, *n* = 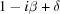, are additive along the X-ray path. In other words, when an X-ray projection is acquired of a two or more compound object the image intensity relates to the contribution from the object along the beam path through a line integral. Therefore without an *a priori* knowledge of the object’s composition or structure a single radiograph is in general not sufficient to reconstruct the spatial distribution of the different materials composing this object. It was shown by Brody *et al.* (1981 ▸) and Lehmann *et al.* (1981 ▸) that some discrimination of materials is feasible if two images are acquired, one with low-energy and the other with high-energy X-rays. They exploit the different dependency on the *Z* number of the cross sections of Compton scattering and the photoelectric effect. This approach is widely used for security applications and also medical purposes using X-ray tube sources (Evans, 2002 ▸). A second approach for dual-energy imaging is to exploit the absorption edge of a selected material in the object and tune the two energies close to it, one below and one above the edge. With this so-called *K*-edge subtraction certain chemical contrast can be achieved. Used for the first time in diagnostics of neurovascular pathology with iodine-containing contrast agent (Rubenstein *et al.*, 1986 ▸), nowdays *K*-edge subtraction is performed mostly with monochromatic X-rays from synchrotron sources in life sciences (Bayat *et al.*, 2001 ▸) and less frequently in materials characterization (Toda *et al.*, 2010 ▸). As compared with the laboratory-based systems there has been little advance using synchrotron sources for single-exposure dual-energy imaging. Most recently, dual-detector approaches were demonstrated (Carnibella *et al.*, 2012 ▸; Rack *et al*., 2013 ▸; Mokso *et al*., 2013 ▸). The harmonic content of a monochromatic synchrotron beam is exploited such that a filter is used between the two detectors to filter out the low-energy spectrum from the X-ray beam that travels to the second detector. This way the first detector closer to the sample registers the image with the first fundamental X-ray energy, while the second detector sees an image with a spectrum corresponding to the higher harmonic of the crystal monochromator. This turns out to be useful for separating bone and soft tissue to reduce artefacts in the image of such mixed attenuation and phase objects typically in small-animal imaging. Yet this method gives a limited space for tuning the energy content of the two images. We show how two images can be acquired simultaneously with fully tunable wavelength in both beam branches. In addition, our new approach offers the advantage of a stereoscopic acquisition (Lee *et al.*, 2011 ▸; Gleber *et al.*, 2009 ▸) pointing towards a new era of fast multidimensional *in vivo* and *in situ* imaging with brilliant X-ray beams.

## 2. Methods   

**Figure 1:**
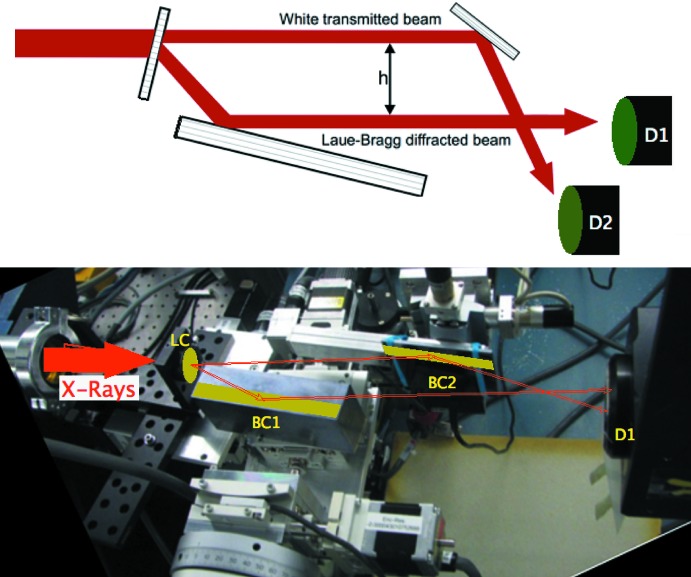
Schematic and experimental crystal setup realised at the TOMCAT beamline. The monochromatic impinging beam is split on the Laue crystal (LC). The two beam branches are diffracted further downstream by the two Bragg crystals (BC1, BC2) before they cross again in the sample plane. The spatially separated images after one and two diffractions are collected at two detector positions (D1 and D2, respectively).

**Figure 2:**
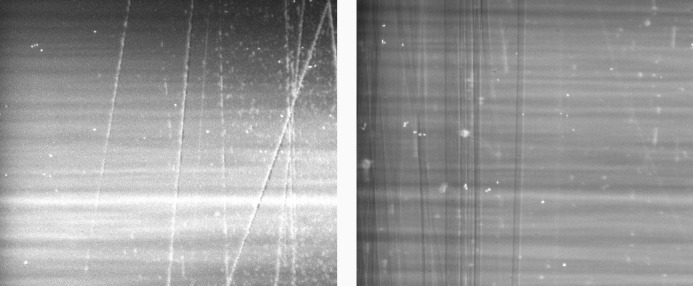
The diffracted beam after the Laue Si crystal and consequently by the Si Bragg crystal (BC1) (left image), and the beam transmitted through the Laue crystal and diffracted by the Si Bragg crystal (BC2) (right image). For the purpose of this visualization the image intensities were normalized. One can see the stripe structure of the multilayer monochromator. The perpendicular lines to the stripes are scratches from the surface processing. Other artefacts are dust on the scintillator.

The concept of tunable dual-energy stereo imaging was previously theoretically described by Oberta & Mokso (2013 ▸). The experiment was performed at the TOMCAT imaging beamline of the Swiss Light Source. The experimental setup shown in Fig. 1 ▸ consisted of a first Laue diffraction crystal and two Bragg diffraction crystals. The function of the Laue crystal was to split the beam into the transmitted beam and the diffracted beam. The transmitted beam can be either monochromatic, as in our case, or polychromatic (when using a white incident beam) to allow the user a broader energy combination. The Laue crystal was a Si crystal with Si(111) planes perpendicular to the crystal surface. The thickness of the crystal was 300 µm; when using a primary incident beam energy of 17.5 keV, the crystal transmission was 65.1%. By rocking the Laue crystal the 17.5 keV beam was diffracted in the horizontal direction to the second Bragg crystal (Fig. 2 ▸, left) and a second diffraction occurred (second beam branch) re-directing the beam to a parallel beam path with the transmitted beam (first beam branch). The transmitted beam impinges on the third Bragg crystal and diffracts under the Bragg angle (Fig. 2 ▸, right). The diffracting Bragg angle is at the same time half of the angle between the two beam branches. They meet at the sample position probing the object from two directions. The detector placed behind the sample in either of the two branches of the beam was a PCO.edge 4.2 coupled to a 300 µm LuAG:Ce scintillator using a 1:1 macroscope. This configuration results in a pixel size of 6.5 µm in all the images shown in this work. For consistency, every radiograph was acquired with an exposure time of 500 ms. The Laue–Bragg beam splitter may be operated in three distinct modes, each representing a distinct imaging method:

(i) In the first configuration (single-energy stereoscopy) the X-ray energy in both branches was set to be equal within the precision of the angular measurement of the crystal tilt. Stereoscopic imaging may be performed with the two beams of the same energy using a detector for each of the two beams transmitted through the sample. At 17.5 keV the stereoscopic angle is 12.6° which is close to the optimal value to subtract volumetric information from only these two projections (Evans, 2002 ▸) and is well suited for three-dimensional particle velocimetry (Lee *et al.*, 2011 ▸).

(ii) In the *K*-edge subtraction mode (Kelcz & Mistretta, 1976 ▸) we select the wavelength (energy) of the two branches close to each other, one just below and one just above the absorption edge of the element for which contrast enhancement is required.

(iii) In dual-energy decomposition mode (Alvarez & Macovski, 1976 ▸) one branch carries photons of low X-ray energy (*e.g.* 20 keV) while the other branch contains high-energy photons (*e.g.* 70 keV) only. The photoelectric effect is dominant in the first while strong Compton scattering will be present in the high-energy image.

**Figure 3:**
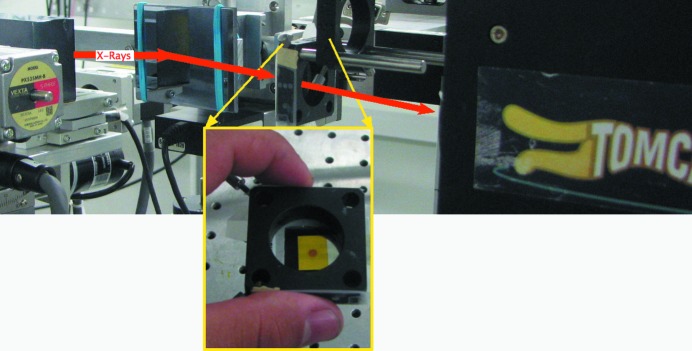
The test sample consisting of a 20 µm Zr foil and a standard copper TEM grid type 400 is placed between the BC2 crystal and the detector.

In the last two modes of operation the energy in the two branches is different while in the first mode both branches are almost at the same wavelength. The quantitative analysis consisted of calculating the effective thickness of the samples using the following rationale. The image intensity is proportional to the number of photons impinging on the detector after transmission through the sample. In our case the two beams are monochromatic which allows the total transmission to be decomposed into the two basis materials (Lehmann *et al.*, 1981 ▸):
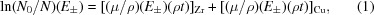
where 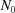 and *N* are the mean number of incident and outgoing photons per pixel, 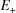 and 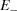 are the two energies that bracket the *K*-edge discontinuity, ρ is the density of the material, *t* is the transmitted path length, 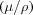 is the mass attenuation coefficient and 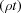 is the mass density, in our case of zirconium and copper (Fig. 3 ▸).

The mass densities of zirconium and copper are calculated by solving this system of two expressions for the two energies,
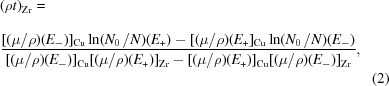


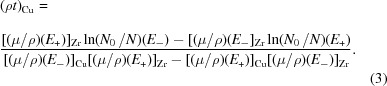



## 3. Results   

We present here results obtained in two modes of operation of the Laue–Bragg beam splitter. In the single-energy stereoscopy mode the two beams probe the sample at the same wavelength but angularly separated by double the Bragg angle of the crystals. These two wavefronts are shown in Fig. 2 ▸, both acquired with the same pixel size of the detector. Choosing a different spatial resolution for each of the two images can be particularly interesting in the case of tomography when, across a single rotation by 180° in parallel beam geometry, two tomograms of different spatial resolution are acquired. This is an alternative to the dual-detector system using semi-transparent optics (Rack *et al.*, 2013 ▸; Mokso *et al*., 2013 ▸). In Fig. 2 ▸ the stripe pattern in the horizontal direction is due to the imperfection of the multilayer monochromator installed upstream of the Laue–Bragg splitter. The vertical scratches are residues from the surface polishing of the Si crystals. The grain size of the polishing material (SiC 320) was 50 µm. These scratches are not visible after the Laue diffraction crystal because the Laue crystal was etched between the grinding and polishing process. The remaining bright spots in the images are due to dust on the scintillator.

In the two other modes of operation the energy in the two branches is different. To operate the Laue–Bragg beam splitter in the *K*-edge subtraction mode we set the X-ray energy of the incident beam to 18 keV using the multilayer monochromator permanently installed at the beamline. The bandwidth of approximately 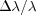 = 10^−2^ offers the possibility to use this setup for simultaneous *K*-edge subtraction imaging. For the dual-energy decomposition mode the incident beam must be polychromatic without using the monochromator.

As a sample we used a zirconium foil of 20 µm thickness with a *K*α absorption edge at 18 keV (17995.9 eV). Attached to the zirconium foil was a copper mesh 400 (Fig. 3) with hole sizes of 45 µm. At the X-ray energy of 18 keV the theoretical transmission of the 20 µm zirconium foil changes from 83% below the edge to 29% above the absorption edge. Copper does not have an absorption edge near 18 keV and therefore the transmission of the copper mesh varies only by about 1% within the bandwidth of 400 eV around the mean energy of 18 keV.

**Figure 4:**
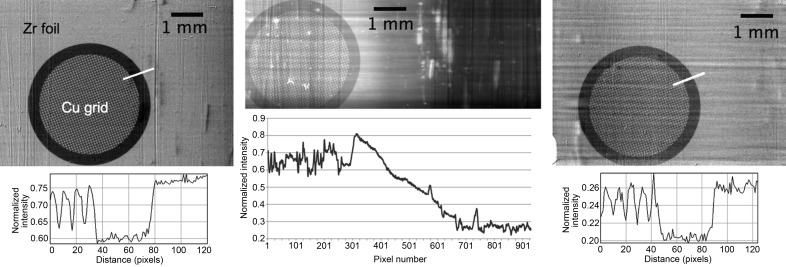
(Left) Image of the Zr mesh below the *K*1 absorption edge, (middle) image of the Zr mesh with one half of the image below and the other half above the *K*1 absorption edge and (right) image of the Zr mesh above the *K*1 absorption edge. A normalized mean intensity profile in the horizontal direction across the whole image is shown in the lower part of the middle image. The normalized intensity profiles shown on the lower left and right plots are captured along the white line at the upper right border of the grid. The length of the profile plots is 120 pixels corresponding to 780 µm to show the contrast in the grating with the hole size of 45 µm. All images are acquired using the same detector configuration with pixel size of 6.5 µm.

By rocking the Si Bragg crystal (BC1) to the low-energy end of the Darwin–Prins curve and the Si Bragg crystal (BC2) to the high-energy end of the Darwin–Prins curve we created two beams with an energy below and above the *K*α absorption edge of zirconium. Fig. 4 ▸ (left) shows an image of the sample below the absorption edge and above the absorption edge (right). The middle part of the figure represents a ‘dual-energy’ image acquired in a configuration when one half of the image is below and one half above the absorption edge. The image with two different energies is possible due to energy dispersion of the crystal arrangement of the Bragg crystal (BC2) accepting a broadband transmitted beam. Rocking the crystal towards the opposite end of the Darwin–Prins curve enables the absorption-edge transmission at a certain point to be seen. The normalized mean intensity profile is shown in the lower part of the images. In the middle image this profile shows the variation of the attenuation by the zirconium foil across the horizontal direction where from left to right the energy increases from 17.9 to about 18.1 keV. The transmission drops from 0.81 (5) to 0.26 (4) from left to right corresponding to crossing the absorption edge of the zirconium. The gradient of this drop is a function of the energy profile and is evaluated in the line profile plot in Fig. 4 ▸ (middle). The line profiles in Fig. 4 ▸ (left and right) depict a small region of 120 pixels each (full image width is 900 pixels or 5.85 mm) to quantify the contrast (visibility) of the grating structure. As expected, the contrast between the copper grid structures on the zirconium background does not change in relative terms.

We performed quantitative analysis of the results using equations (2) and (3). First we determined the precise thickness of the zirconium foil to be 23 µm instead of the nominal value of 20 µm as given by the producer. Secondly the thickness of the copper TEM grid was calculated to be 5 µm. In this demonstration the sample stage did not allow rotation of the sample to perform tomography; therefore, we chose this planar sample that demonstrates the basic properties of the proposed system.

## 4. Conclusions   

We have experimentally realised for the first time a broadly tunable dual-energy stereo imaging system (in our case the energy range is 8 to 30 keV). Using a broadband illumination from a synchrotron source as the incident beam, two projection images of a test sample were recorded each at an independently selected X-ray energy. In a special case both the higher and the lower energy of the Darwin–Prins curve may be present in the same image spatially separated. Not only could the contrast in the images be increased this way but a quantitative assessment of the sample composition was also demonstrated. An advantage of the presented design as compared with earlier works (Carnibella *et al.*, 2012 ▸; Elleaume & Bravin, 1999 ▸; Suortti *et al.*, 1993 ▸) is threefold: (i) a large range of wavelength separation between the two beams, (ii) easy tuning of the X-ray wavelength between the two beams and (iii) simultaneous acquisition of two angularly separated projections. As such the instrument we present is the only one to our knowledge that can be used for *K*-edge subtraction, dual-energy decomposition and single- or dual-energy stereoscopic imaging. The switching between these methods consists essentially only of adjusting the pitch of one crystal in the Bragg condition. We therefore expect that the ease of using this design will trigger a wider use of dual-energy imaging of dynamic systems. The transition from this demonstrator to a mature system will consist of enabling sample rotation and performing tomography. To optimize for efficiency, multilayers may be used instead of the Bragg crystals. In addition to the dual-energy mode, we propose three-dimensional studies using the same energy in both branches resulting in two distinct Fresnel diffraction patterns. These will be an input to retrieve the phase shift of the X-rays interacting with the sample. The multitude of configurations of the proposed system especially in 3D should be further exploited in the future.
